# VGF Peptide Profiles in Type 2 Diabetic Patients’ Plasma and in Obese Mice

**DOI:** 10.1371/journal.pone.0142333

**Published:** 2015-11-12

**Authors:** Filomena D’Amato, Barbara Noli, Laura Angioni, Efisio Cossu, Michela Incani, Irene Messana, Barbara Manconi, Paola Solinas, Raffaella Isola, Stefano Mariotti, Gian-Luca Ferri, Cristina Cocco

**Affiliations:** 1 Department of Biomedical Sciences, University of Cagliari, 09042, Monserrato, Italy; 2 Department of Medical Sciences, University of Cagliari, 09042, Monserrato, Italy; 3 Department of Life and Environmental Sciences, University of Cagliari, 09042, Monserrato, Italy; GDC, GERMANY

## Abstract

To address the possible involvement of VGF peptides in obesity and diabetes, we studied type 2 diabetes (T2D) and obese patients, and high-fat diet induced obese mice. Two VGF peptides (NAPP-19 and QQET-30) were identified in human plasma by HPLC-ESI-MS. The VGF C-terminus, the above two cleaved peptides, and the TLQP-21 related peptide/s were studied using ELISA and immunohistochemistry. In euglycemic patients, plasma NAPPE and TLQP like peptides were significantly reduced with obesity (74±10 *vs*. 167±28, and 92±10 *vs*. 191±19 pmol/ml, mean+SEM, n = 10 and 6, obese *vs*. normal BMI, respectively, p<0.03). Upon a standard glucose load, a distinct response was shown for VGF C-terminus, TLQP and QQET-like (ERVW immunoreactive) peptides in euglycemic normal BMI patients, but was virtually abolished in euglycemic obese, and in T2D patients independently of BMI. High-fat diet induced obese mice showed reduced plasma VGF C-terminus, NAPPE and QQET-like (ERVW) peptide/s (3±0.2 *vs*. 4.6±0.3, 22±3.5 *vs*. 34±1.3, and 48±7 *vs*. 100±7 pmol/ml, mean+SEM, n = 8/group, obese *vs*. slim, respectively, p<0.03), with a loss of the response to glucose for all VGF peptides studied. In immunohistochemistry, TLQP and/or VGF C-terminus antibodies labelled VGF containing perikarya in mouse celiac ganglia, pancreatic islet cells and thin beaded nerve fibres in brown adipose tissues, with fewer in white adipose tissue. Upon the glucose load, tyrosine hydroxylase and VGF C-terminus immunoreactive axons became apparent in pancreatic islets of slim animals, but not in obese animals. Alltogether, a significant loss of VGF peptide immunoreactivity and/or their response to glucose was demonstrated in obese patients, with or without T2D, in parallel with a similar loss in high-fat diet induced obese mice. An involvement of VGF in metabolic regulations, including those of brown and/or white adipose tissues is underlined, and may point out specific VGF peptides as potential targets for diagnosis and/or treatment.

## Introduction

Type 2 diabetes (T2D) is a chronic disorder of carbohydrate, fat and protein metabolism, steadily increasing worldwide. The global epidemic of T2D appears to be largely secondary to insulin resistance induced by obesity [1]. Of metabolically healthy adults, those with body mass index (BMI) ≥ 30 kg.m^-2^ show an increased risk of developing T2D, compared to normal weight individuals [2–4]. Adipose tissue appears to be a critical component in metabolic control, responding to nutritional, hormonal and neuronal signals [5, 6] by releasing hormones including adipocytokines, which in turn regulate insulin sensitivity [7].

The neurotrophin responsive gene *vgf* (nonacronymic name) has recently been shown to be involved in the regulation of energy balance [8, 9]. VGF mRNA is selectively expressed in neurons and neuroendocrine elements, and its primary translation product, the VGF protein, gives rise to several low molecular weight VGF peptides [10, 11]. These are stored in secretory vesicles and can be secreted upon stimuli [12, 13]. One such naturally occurring VGF peptide, TLQP-21, was shown to increase resting energy expenditure upon intracerebroventricular injection [14]. Such peptide appears to be present in sympathetic nerve fibres in WAT, to bind at high affinity to adipocyte membranes, and to increase lipolysis *via* activation of noradrenaline/β-adrenergic receptor pathways [15, 16]. Chronic administration of TLQP-21 delayed the onset of overt diabetes by preserving islet cell mass in Zucker Diabetic Fatty rats [17]. The C-terminally extended peptide named TLQP-62 distinctly stimulated basal insulin secretion in several insulinoma cell lines [18]. A further peptide derived from a different portion of VGF, named NERP-2, was found to increase glucose-stimulated insulin secretion from the β-cell line MIN6, as well as from isolated mouse pancreatic islets [19]. Further complexity, as well as possible diverging roles of yet uncharacterized VGF peptides are suggested by the remarkable phenotype of *vgf* knockout mice, which are hyperactive and hypermetabolic, with a deranged hypothalamic response to feeding [8, 20]. None the less, only a few studies have so far addressed human obesity and/or diabetes. In a group of patients treated for idiopathic intracranial hypertension, VGF immunoreactivity proved higher in cerebro-spinal fluid from obese, compared to non-obese subjects [21]. The number of neurons expressing both NPY and VGF was increased in the hypothalamic infundibular nucleus, but decreased in the nucleus of tractus solitarius of T2D patients, compared to non-diabetic controls [22].

To address the potential role of VGF in obesity and T2D, we studied a mouse model of high-fat diet induced obesity, in parallel with human newly diagnosed diabetics and age-matched euglycemic controls, classified according to their body mass index (BMI). Four VGF peptides were investigated, including the TLQP peptides which exert known actions on metabolic regulations [14, 15, 18].

## Material and Methods

### Human studies

Patients (20–81 years, male, BMI 19–47 Kg m^-2^) underwent a standard oral glucose tolerance test (OGTT, with 75 g glucose), according to the 2013 American Diabetes Association recommendations [23], because of risk factors for T2D (obesity, hypertension, dyslipidaemia and/or T2D in first degree relative/s). Plasma samples were aliquotted at the time of OGTT, and stored frozen at -80°C. All subjects were classified as: “euglycemic”, or “T2D”. A novel diagnosis of T2D was established when plasma glucose (measured using a Miura 200 analyser, ISE, Italy) was either: >7.0 mmol/L (126 mg/dL) while fasting, or: >11.1 mmol/L (200 mg/dL) at 120 min after the glucose load. Both T2D and euglycemic subjects were further subdivided in age-matched groups according to their BMI, as follows: “normal weight” (BMI <24.9 Kg m^-2^; N = 6 and 6, respectively), “overweight” (25<BMI<29.9; N = 6 and 7, respectively), or “obese” (BM>30 Kg m^-2^; N = 8 and 10, respectively). Of 43 subjects examined, 21 (50%) showed arterial hypertension, 14 (33.3%) hypercholesterolemia with/without associated hypertriglycerideremia. Arterial hypertension and dyslipidemia were similarly distributed across all subgroups examined. Plasma insulin was measured by RIA (DIAsource ImmunoAssays S.A., Belgium). Human samples were collected between 2010 and 2013 at the Endocrinology and Diabetes Unit, Department of Medical Sciences, University of Cagliari. All participants provided their written informed consent, and the study were approved by the Ethical Committee of Cagliari AOU (“Azienda Ospedaliera Universitaria”), protocol n. 450/09/C.E.

### High-resolution HPLC-ESI-MS and MS/MS analysis

Human plasma was pooled from 16 euglycemic subjects (about 1 ml each), filtered through a 30kDa cutoff Amicon Ultra device (Merck Millipore, Tullagreen Carrigtwohill Co. Cork, Ireland), dried using a Vacufuge Concentrator (Eppendorf, Milan, Italy), redissolved in 0.5 mL of 0.1% trifluoroacetic acid (TFA) and analysed by high-resolution HPLC-ESI-MS using an Ultimate 3000 HPLC (Dionex, Sunnyvale, CA, USA) equipped with a FLM-3000-Flow manager module, and an LTQ Orbitrap XL (Thermo Fisher). A 300SB-C18 Zorbax column (5 μm, 300 Å pore size, 150 × 1.0 mm: Agilent Technologies, Santa Clara, CA) was run with 0.056% aqueous TFA (eluent A) and 0.050% TFA in acetonitrile/water (80:20 v/v: eluent B). A step gradient was applied as follows: (i) 5 to 55% B, 40 min; (ii) 55 to 100% B, 8 min; (iii) 100% B to 5% (9 min), at a flow rate of 80 μL/min. The injection volume was 20 μL. Positive MS/MS spectra were recorded in full scan mode using the lock mass for internal mass calibration (polydimethylcyclosiloxane, 445.1200 *m/z*) with a 60000 and 30000 resolutions, respectively, and a 350 to 2000 *m/z* range. The three most intense multiply charged ions were fragmented using collision-induced dissociation (35% normalized collision energy). Tuning parameters were: capillary temperature 250°C, source voltage 4.0 kV, capillary voltage 48 V, tube lens voltage 170 V. High-resolution MS/MS were annotated manually, as well as using the Proteome Discoverer 1.2.0 software (2010, Thermo Fisher Scientific) based on SEQUEST cluster as search engine (University of Washington, Seattle, WA, licensed to Thermo Electron, San Jose, CA) against the UniProt Knowledgebase (release 2014–08, 03-Sep-2014).

### Animal studies

Male CD1 mice (Charles River, Lecco, Italy: 4–5 weeks, 22–26 g body weight), were housed under standard conditions (23±2°C, 60±10% relative humidity, 12–12 h light-dark cycle, 6 per cage), and fed standard chow (3.9 Kcal/g, 10.7% from fat, code 4RF21 from Mucedola, Italy; N = 24 mice), or a high-fat diet consisting of the same chow with the addition of 30% w/w porcine lard (from a local supplier; 5.6 Kcal/g, 45% from fat; N = 24 mice). All animals had free access to water and food throughout. At 16 weeks, all high-fat diet mice showed a body weight higher than the average of the lean group +2 SD, hence were deemed “obese”. Both “slim” (N = 24) and “obese” mice (N = 24) were fasted (8 hours) and further subdivided into a: “glucose load”, and a: “fasting” group (N = 12+12 each). Mice underwent blood sampling from the tail vein (for rapid glucose testing: multiCare-in, Biochemical Systems International, Arezzo, Italy), then were either sacrificed immediately (“fasting” group), or received a glucose load (3g/kg, *i*.*p*.) and were sacrificed after 120 min (“glucose load” group). At sacrifice, mice underwent deep diethyl ether anaesthesia, blood was drawn from their right heart (with ethylenediaminetetraacetic acid, 1.78 mg/mL), hence were either perfusion fixed (for immunohistochemistry, N = 4 per each of the four resulting groups: with 40 g/L paraformaldehyde in 0.1 mol/L PO_4_, *via* the left ventricle), or were rapidly dissected (N = 8 per group) for tissue VGF peptide extraction. Blood samples were rapidly spun, and the resulting plasma was aliquotted and stored frozen. Fixed tissue samples (celiac ganglion, pancreas, epididymal and interscapular fat) were rinsed in PBS containing 70 g/L sucrose and 0.1 g/L NaN_3_, set up in blocks using cryoembedding media [24] and snap-frozen. Cryosections (5–30 um) were collected on slides coated with poly-l-lysine (Sigma, Milan, Italy). For tissue VGF peptide assays, WAT and BAT were collected on ice and extracted as previously described [25]. Experimental protocols were approved by the Ethical Committee of the University of Cagliari and were performed in agreement with Italian legislation, and the care and use of animals approved by the American Physiological Society and EEC Council Directive of 24 November 1986 (86/609).

### VGF antibodies

Antisera (Table 1) were produced as previously described [26, 27]. Briefly, a His_615_-Arg_616_-Pro_617_ sequence is found at the C-terminal end of rat and mouse VGF, as opposed to Arg_613_-Arg_614_-Pro_615_ in man [28], and antisera were raised against the corresponding nonapeptides conjugated *via* an N-terminal tyrosine. TLQP-peptides were isolated from rat brain and proved to be cleaved from VGF at the rat VGF_553-555_ (Arg_553_-Pro_554_-Arg_555_) processing site [29], at least their N-terminal five amino acids (Thr-Leu-Gln-Pro-Pro) being identical in human and rat VGF. The rat VGF_556-565_ peptide was synthesized and conjugated at its C-terminus. Two further, recently identified peptides appeared to correspond to rat VGF_489-507_ and VGF_180-209_ (or identical sequences within human VGF), and were named: NAPP-19 (tentatively NERP-4), and QQET-30 (tentatively NERP-3), respectively [30]. Synthetic decapeptides at the N-terminal end of NAPP-19 (rat VGF_489-497_), and at the C-terminal end of QQET-30 (rat VGF_200-209_) were conjugated to either keyhole limpet haemocyanin, or bovine thyroglobulin, *via* an additional Cysteine residue at their C- and N-terminus, respectively, and used for immunizations.

**Table 1:** VGF antibodies and ELISA characterization.

native	peptide	antigen	used (AG)	antibody	short name	expected reactivity	coating /	IC_50_	CV1	CV2	cross
				specificity			standard	pmol/mL	%	%	react.
							peptide				%
AQEE-30	r VGF_588-617_	r VGF_609-617_	–Y–IEHV LLHRP	C-term	r/m VGF C-term	fragments with AG	**r VGF**_**609-617**_ *	**0.02**	**3**	**4**	**100**
						sequence at C-term,	h VGF_607-615_				<0.01
						e.g. VGF,					
						peptide V, TLQP-62,	h VGF_603-612_				<0.01
						VGF20, etc [29]					
peptide V ^**1**^	h VGF_586–615_	h VGF_607–615_	–Y–IEHVLLRRP	C-term	h VGF C-term		**h VGF**_**607-615**_ *	**10**	**6**	**8**	**100**
						As above	h VGF_603-612_				0.8
							r VGF_609-617_				0.4
TLQP-21	r VGF_556-576_	r VGF_556-565_	TLQPP ASSRR–C–	N-term	TLQP	TLQP-11, TLQP-21,	**r VGF**_**556-564**_ *	**1.1**	**4**	**6**	**100**
						TLQP-62 [32]	r VGF_556-566_				122
							r VGF_556-576_				183
							r VGF_555-564_ ^**2**^				3.5
							h VGF_554-574_				60
							h VGF_554-577_				65
NAPP-19 ^4^ ^,^ ^6^	r VGF_489-507_	r VGF_489-497_	NAPPEPVPP–C–	N-term	NAPPE	NAPP-19, peptides	**r VGF**_**489-497**_ *	**6**	**5**	**8**	**100**
	h VGF_485–503_					extended at C-term,	r VGF_489-507_				95
						e.g. VGF20 [29]	r VGF_488-496_ ^**2**^				<0.001
QQET-30 ^5^ ^,^ ^6^	r VGF_180-209_	r VGF_200–209_	–C–LESPGPERVW	C-term	ERVW	QQET-30	**r VGF**_**197-206**_ *	**10**	**8**	**10**	**100**
	h VGF_177–206_					(forms extended at	r VGF_197-207_ ^**3**^				<0.001
						N-terminus?)	h VGF_177-206_				90
							h VGF_199-206_				98

IC50: dose producing 50% inhibition; CV1 and CV2: intra and inter assay variation, respectively; cross-react.: cross reactivity; h: human, r: rat (for all peptides studied, rat and mouse sequences are identical); C- / N-term: C- / N-terminus, respectively

^*^ used for plate coating and measurement standard

^1^ identified in bovine pituitary, identical to the corresponding human AQEE-30 sequence

^2–3^ include: ^2^ an N-terminal Arg / Lys residue, as appropriate, or: ^3^ a C-terminal Arg residue (first amino acid adjacent to peptide within the corresponding cleavage site), to address possible cross-reactivity with N- or C-terminally extended forms, as relevant

^4–5^ tentatively named: NERP-3 and NERP-4, respectively

^6^ an identical amino acid sequence is present in human and rat.

Thus, our QQET-30 antibodies were produced against its C-terminal peptide C-LESPGPERVW, and will be referred to as: “ERVW” antibodies (or: “ERVW” ELISA and “ERVW” like peptide/s, where appropriate). All antibodies we produced against VGF peptides were intended to be specific for the corresponding truncated sequence at their respective N- or C-terminus (Table 1), hence are expected to cross-react extensively with peptides elongated at their other end. Specificity was addressed by ELISA (Table 1), as well as by immunohistochemistry, the relevant immunostaining being prevented by pre-absorption of each antiserum with the corresponding peptide (up to 100 nmoL/mL) in each case. VGF C-terminus and TLQP antibodies have been characterized previously, and were found to recognize a number of VGF peptides of different MW containing their corresponding C- and N-terminal peptide sequence used for immunization, respectively [25, 31, 32]. In view of the methods used, the expression: VGF C-terminus peptide/s, or: TLQP peptide/s, etc. will be intended to indicate the corresponding: “peptide-like immunoreactivity” throughout.

### Immunohistochemistry

Cryosections were incubated overnight in a humid chamber, with primary antibodies diluted in PBS containing 30ml/L of normal donkey serum and 30 ml/L of normal mouse serum, and 0.02 g/L NaN_3_. Double immunofluorescence experiments were carried out mixing one VGF antibody (dilution: 1:300–3000, as appropriate), with either a tyrosine hydroxylase (TH, from Sheep, Chemicon, Temecula, CA), or an insulin antibody (from Guinea Pig, Biodesign, Saco, MA). The relevant species-specific donkey secondary antibodies, conjugated with either Cy_3_ or Cy_2_ (Jackson Immunoresearch Laboratories, West Grove, PA) were used to reveal primary antibody binding sites. Slides were coverslipped with PBS-glycerol, observed and photographed using BX41 and BX51 fluorescence microscopes (Olympus, Milan, Italy), equipped with simultaneous visualization filter sets developed with Chroma (Bellows Falls, VT), and with Fuji S2 and S3 Pro digital cameras (Fujifilm, Milan, Italy). Routine controls included substitution of each antibody, in turn, with PBS, the use of pre-immune or non-immune sera, and the testing of each secondary antibody with their respective non-relevant primary antibodies.

### Enzyme linked immunosorbent assay (ELISA)

VGF assays and their characterization are summarized in Table 1. Competitive ELISA was performed as previously reported [25, 27]. Briefly, multiwell plates (Nunc, Milan, Italy) were coated with the relevant synthetic peptide (3 h, at room temperature), hence treated with PBS containing 9% normal donkey serum, 20 nM aprotinin, and 1 mg/mL ethylenediaminetetraacetic acid (EDTA) for 2 hours. Primary incubations were carried out in duplicate, including serial dilutions of standard (0.005–500 pmol/ml) or samples (4 h at room temperature). Biotinylated secondary antibodies (Jackson, West Grove, PA), streptavidin-peroxidase conjugate (Biospa, Milan, Italy), and a tetrametylbenzidine substrate (TMB X-traKem-En-Tec, Taastrup, Denmank) were used to reveal bound antibodies. The reaction was stopped with HCl (1 mol/L) and the optical density was measured at 450 nm using a multilabel plate reader (Chameleon: Hidex, Turku, Finland). Recovery of synthetic peptide/s added to plasma, or to tissue samples at extraction was >85% for all assays used. Absolute numerical values of tissue concentrations measured *per* each peptide studied ought to be considered (or compared across different peptides) with caution, pending precise characterization of the heterogeneous molecular species present.

### Statistical analyses

Analysis of variance was carried out by one-way ANOVA, followed by *post hoc* tests (Student-Newman-Keul tests), while the paired two-tailed Student’s t-test was used for two sample comparisons, by means of the StatistiXL software in both cases. In human subjects, correlations between serum VGF peptides and biochemical parameters was analyzed by non-parametric methods (Mann-Whitney and Krskall-Wallis tests).

## Results

### HPLC-ESI-MS of human plasma

**Fig 1:**
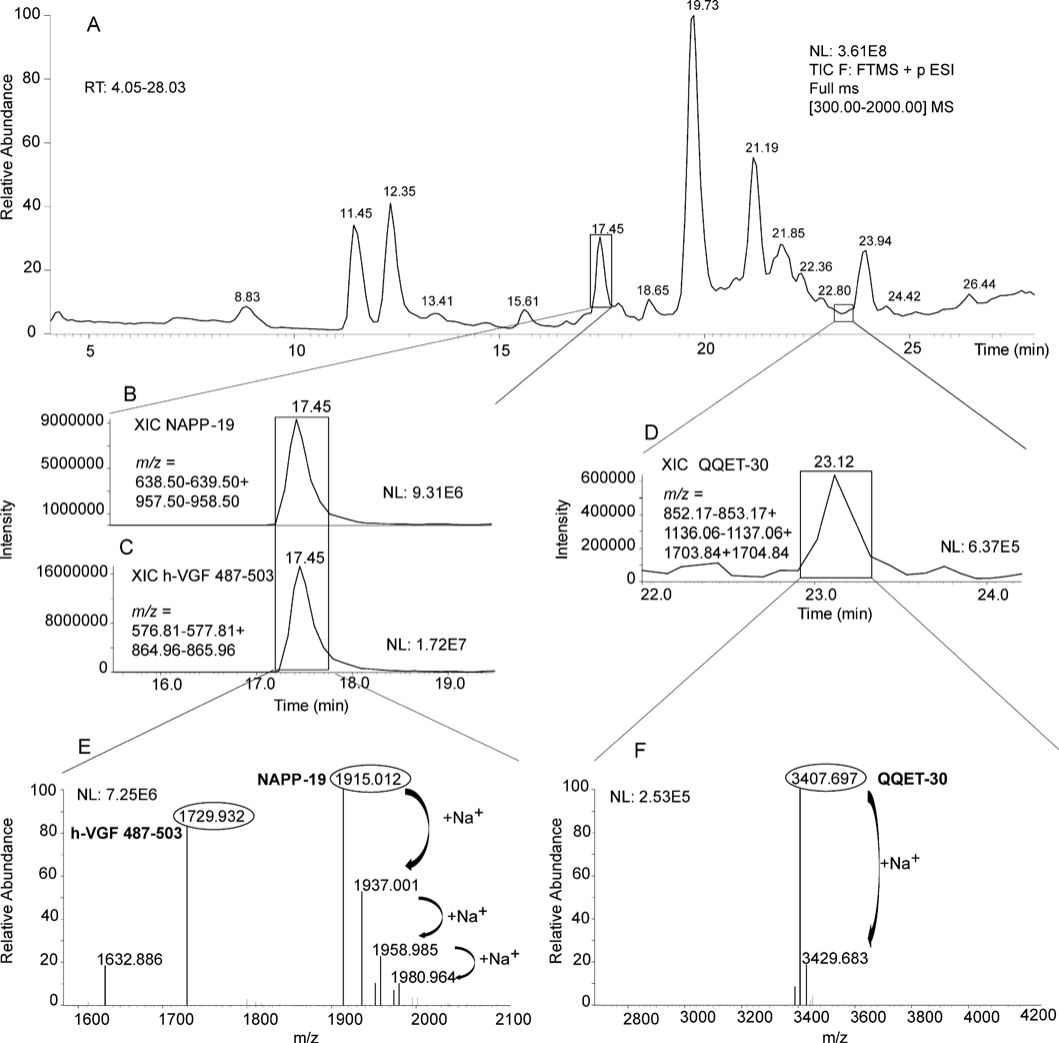
HPLC-ESI-MS of human plasma. The NAPP-19 and QQET-30 peptides were unambiguously identified. Panel A: HPLC-ESI-MS total ion current (TIC) profile, with enlarged view (range 4.05–28.03 min) of the human sample (<30kDa preparation). Panels B-D: extracted Ion Current (XIC) peaks corresponding to NAPP-19 (i.e. VGF_485-503_: B), its truncated co-eluting form VGF_487-503_ (C), and QQET-30 (D). The same panels indicate m/z values of the multiply charged ions used to extract the ion current peaks. Panel E: deconvoluted ESI-MS spectra of NAPP-19 (monoisotopic [M+H]^+^ at 1915.012 m/z) and VGF_487-503_ (monoisotopic [M+H]^+^ at 1729.932 m/z). Panel F: deconvoluted ESI-MS spectrum of QQET-30 (monoisotopic [M+H]^+^ at 3407.697 m/z). Sodium ion adducts are also depicted (ibidem). RT = retention time; NL = normalization level; amino acid numbering is referred to human VGF throughout.

Since little is known as to the precise identity of VGF peptides in peripheral human blood, we tested a pool of euglycemic subject plasma samples, treated to select <30kDa molecular species. The peptides NAPP-19 (human VGF_485-503_, also named: NERP-4) and QQET-30 (human VGF_177-206_, also named: NERP-3) were unambiguously identified (Fig 1), with the addition of a two-amino acid shorter form related to NAPP-19 (human VGF_487-503_). The analysis of MS/MS spectra confirmed the sequence of both peptides (see annotated HPLC-ESI-MS/MS spectra in S1 and S2 Figs). The latter attribution was based on the very close agreement between experimental and theoretical m/z values of [M+H]^+^ (theoretical 3407.69 m/z, Fig 1F), as well as on the correspondence of the relevant elution time with the one of synthetic QQET-30 peptide.

### ELISA of human plasma

**Fig 2:**
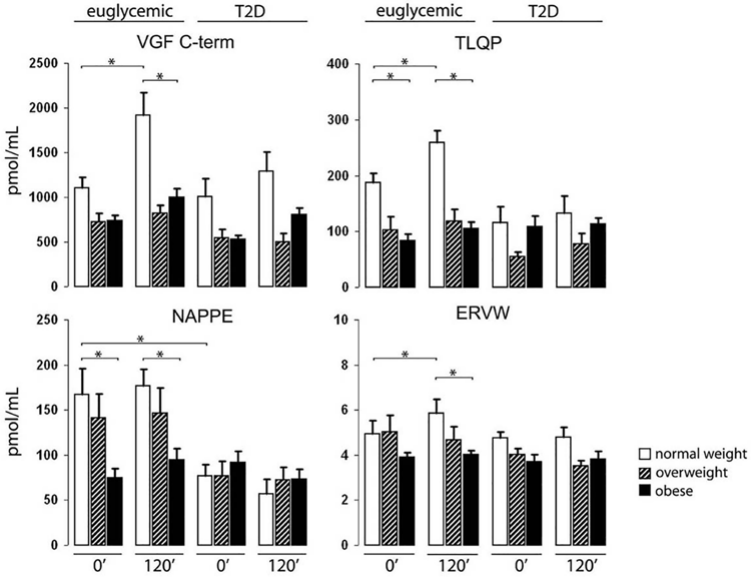
VGF peptides in human plasma. VGF peptides were studied at fasting (0’), and upon an oral glucose tolerance test (120’). Euglycemic and newly diagnosed type 2 diabetic subjects (T2D) were subdivided in: normal weight, overweight and obese. Normal weight, fasting euglycemic patients showed significantly higher TLQP and NAPPE immunoreactive peptide/s *vs*. the corresponding obese subjects (first set of bars in each panel), as well as *vs*. normal weight diabetics (NAPPE peptide/s only, third *vs*. first set of bars). Upon the glucose load, VGF C-terminus, TLQP and ERVW peptide/s were stimulated in euglycemic normal weight subjects, but not in the other body weight groups (second *vs*. first set of bars), nor in diabetic patients (fourth *vs*. third set of bars). VGF C-term: VGF C-terminus peptide/s, ERVW refers to the C-terminally directed assay used for QQET-30 like peptide/s (see Table 1); means±SEM, * = P<0.03.

Measurable immunoreactivity was found for all VGF peptides studied (Fig 2). On the whole, VGF peptides were distinctly more abundant in euglycemic subjects, especially in the normal weight group. In the latter group, TLQP and NAPPE peptide/s were significantly higher than in corresponding obese, as well as (NAPPE peptide/s only) compared to normal weight diabetics. Upon the oral glucose load, immunoreactivity for three out of four VGF peptides showed a significant response in euglycemic subjects, largely in the normal weight group (Fig 2, first two sets of bars: VGF C-terminus, TLQP and ERVW peptide/s). Hence, upon the glucose load the difference between normal weight and obese euglycemic patients became apparent for all peptides studied. T2D subjects failed to show any significant response of any of the VGF peptides studied upon the glucose load (Fig 2, last two sets of bars). No correlation was found between the concentrations of any of the VGF peptides studied and either: age, arterial hypertension, dyslipidemia, or plasma insulin (data non shown).

### ELISA of mice plasma and tissues

**Fig 3:**
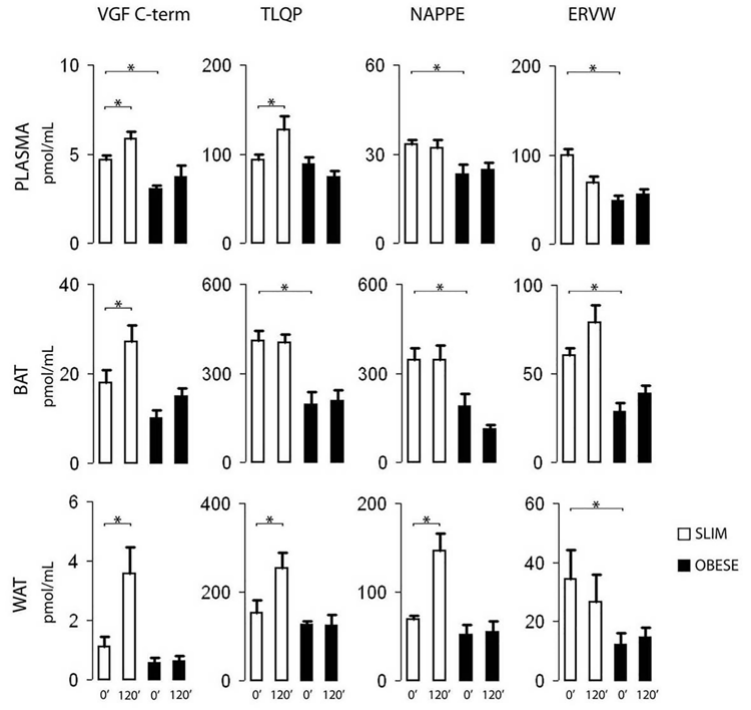
ELISA of VGF peptides in mouse. Upper panel (plasma): upon fasting (0’), higher VGF C-terminus, NAPPE, and ERVW immunoreactive peptide/s were shown in slim *vs*. obese mice, while the glucose load (120’) led to increased VGF C-terminus and TLQP peptide/s in slim mouse, with little response in the obese group. Middle panel (brown adipose tissue, BAT): TLQP, NAPPE and ERVW peptides were higher during fasting (0’) in slim mice, which also showed a distinct increase of VGF C-terminus peptide/s after the glucose load (120’). Lower panel (white adipose tissue, WAT): in basal conditions (0’) ERVW peptide/s levels were higher in slim mice, while the glucose load (120’) resulted in a response of all other VGF peptides in slim mice (only). VGF C-term: VGF C-terminus peptide/s; ERVW refers to the C-terminally directed assay used for QQET-30 like peptide/s (see Table 1); mean±SEM, * = P<0.03.

VGF peptide/s were measured in plasma and in adipose tissues (both BAT and WAT). In basal conditions, plasma levels of VGF C-terminus, NAPPE, and ERVW peptide/s were significantly higher in slim *vs*. obese mice (Fig 3, top panel, 0’ bars). Plasma VGF C-terminus and TLQP like peptides showed a distinct response after the glucose load in slim mice, which was virtually lost in obese animals (ibidem, 120’ *vs*. 0’ bars). In BAT, TLQP, NAPPE and ERVW peptide/s were significantly higher in fasting, slim animals (Fig 3, middle panel, 0’ bars), while VGF C-terminus like peptide/s showed a distinct increase upon the glucose load (ibidem, 120’ *vs*. 0’). Conversely, in WAT (Fig 3, bottom panel) ERVW peptide/s (only) were distinctly higher in fasting, slim *vs*. obese mice, while all other VGF peptides we investigated revealed a significant response to the glucose load, again in slim animals only.

### Immunolocalisation in mice

**Fig 4:**
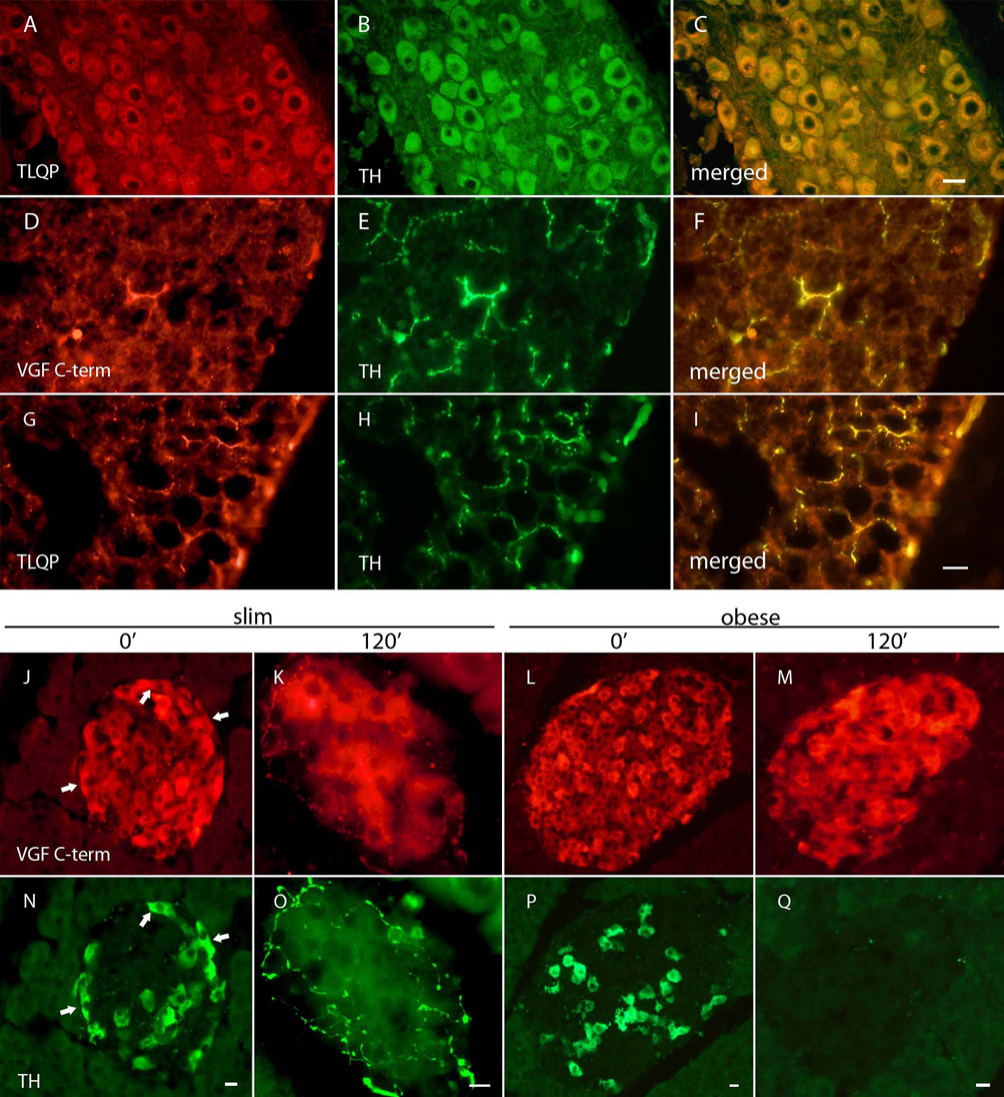
Immunolocalization of VGF peptides in mouse tissues. A-C: In celiac ganglia from slim mice, TLQP antiserum (A) stained a large number of cell bodies, which also contained TH (B). D-I: In BAT from slim mice, VGF-immunoreactivity was shown in thin, beaded nerve axons running close to adipocytes, most of which also labelled for TH (E). TLQP-labelled fibres (G) were more numerous than VGF C-terminus reactive ones (D), and appeared to form a major portion of TH-containing axons. J-Q: In the pancreas from fasting, slim mice (J: time 0’) VGF C-terminus peptide/s were shown in fairly numerous islet cells, including virtually all TH containing cells (N). Upon *i*.*p*. glucose load, distinct TH, as well as VGF C-terminus immunoreactive axons became apparent, running close to and inside the islets (slim mice: K,O *vs*. J,K), while endocrine cells showed reduced staining intensity. Such nerve labelling was virtually lost in obese mice (M,Q at time 120’), while a comparable reduction in islet cell labelling was revealed. VGF C-term: rat/mouse VGF C-terminus antibody, TH: tyrosine hydroxylase, merged: simultaneous dual-visualization of red and green labelling; arrows: examples of dual immunolabelled cells: Cy3, green labelling, Cy2; scale bars: A-C = 10 um, D-I: 50 um, J-Q = 10 um.

In the celiac ganglion (Fig 4A–4C), TLQP antibodies labelled numerous neuronal perikarya, also displaying TH immunoreactivity (Fig 4B), while the other VGF peptides studied showed a comparable though less intense immunostaining. No clear cut differences were observed in the latter location across the four mice groups (not shown). A small number of VGF C-terminus reactive axons (Fig 4D), as well as brighter TLQP labelled fibres were shown in BAT (ibidem: G), where they formed a major subpopulation of TH containing fibres (ibidem: D-I). Similar, though less numerous VGF-immunoreactive axons were found in WAT (not shown).

In fasting, slim mouse pancreas VGF antisera stained a large number of islet cells, as previously reported [26]. The brightest immunostaining was found for the VGF C-terminus antiserum (Fig 4J), which also labelled β-cells reactive for TH (ibidem: J-N, arrows).

After the glucose load (time 120’), TH and VGF immunolabelling of β-cells was somewhat reduced in both slim (ibidem: O) and obese mice (ibidem: Q), while a striking number of TH containing, beaded nerve axons became apparent running close to and inside islets and also displaying VGF C-terminus immunoreactivity (ibidem: K,O). The latter labelled nerves were confined to slim mice, being undetectable in obese animals (ibidem: M,Q).

## Discussion

Our study reports the novel identification of two VGF derived peptides in the human plasma, the distinct response of several VGF peptides to an oral glucose load (OGTT) in normal weight subjects, and their down regulation and blunted response in obese subjects and in T2D patients. Experiments on high fat diet induced obese mice largely confirmed the latter findings.

### Nomenclature of VGF peptides and VGF antibodies / ELISA

A prevalent approach for the naming of VGF peptides has been the use of the single-letter codes of the first four amino acids (at the peptide N-terminus), followed by the number of amino acids residues (e.g. TLQP-21). We used such peptide denomination, appending further information such as their sequence within VGF, or other denominations.

Apart the VGF C-terminus, we aimed at distinguishing cleaved VGF peptides, as opposed to the same sequences embedded within larger molecular forms encompassing the relevant cleavage site/s. For instance, we aimed at the “free” (cleaved) TLQP-21 / TLQP-62 N-terminus (sequence: **TLQPP**ASSRR…), *vs*. the identical sequence contained internally (…PNYI-RPR-**TLQPP**ASSRR…). Thus, antibodies and ELISA assays were named (Table 1) on the basis of the first few amino acids at the N- or C-terminal sequence used for immunizations, to underline antibody and ELISA specificity (e.g.: ERVW antibodies, directed to the C-terminus of the QQET-30, or NERP-3 peptide). Minimally extended peptides encompassing the closest amino acid residue within the relevant cleavage site (e.g. **R-TLQPP**ASSRR…) were used to gain insight as to antibody selectivity (Table 1), though the minimal difference involved *vs*. the immunogen predictably resulted in distinct over-estimation of potential cross-reactivity with native peptides present *in vivo*.

Unavoidably, antibodies highly selective for the N-, or C-terminus of a peptide will be unable to distinguish molecular forms extended at the other end (C- or N-terminus, respectively). As mentioned, our ELISA data represent the overall immunoreactivity resulting from the heterogeneous molecular forms present, which share the truncated sequence for which the assay is specific. The complexity of VGF peptide molecular forms has been the subject of several molecular and proteomic studies [21, 29, 30], and the samples and tissues we studied are most likely to have contained different molecular species related to the VGF immunoreactivities we analysed. Pending their precise identification, major molecular forms expected to cross-react in our assays are briefly summarized in Table 1. In view of the pathophysiological implication of molecular species of VGF peptides which are, or may be, endowed with differing bioactivity and role/s, further focused studies are warranted.

### VGF peptide identification in human plasma

In human plasma, HPLC-ESI-MS identified the authentic VGF peptides QQET-30 (also named NERP-3) and NAPPE-19 (also named NERP-4). So far, little other information is available as to the precise identity of circulating VGF peptides. Using immunochemical methods, several VGF peptides were studied in rat plasma upon osmotic stimuli [10] and during the estrous cycle [33], while the VGF protein was assessed in SOD1 G93A transgenic mice [34]. Our present inability to detect other VGF peptides is perhaps not unexpected in view of the molecular complexity of plasma. In fact, a variety of issues are yet to be addressed, including possible binding to plasma proteins, while the known molecular heterogeneity of VGF peptides [28, 35] may have resulted in a relatively low abundance of single MW species.

### ELISA of VGF peptides in plasma

Measurable immunoreactivity was found for all VGF peptides studied, in both human and mouse. The number of human subjects studied was small, within the framework of a pilot study. None the less, in both species studied euglycemic slim subjects showed a distinct response to the glucose load, or higher fasting levels in slim subjects / animals *vs*. obese or T2D. Similarly, in both species the distinct response of (at least) plasma VGF C-terminus and TLQP peptides found in euglycemic slim subjects, or mice was virtually lost in euglycemic obese and in all T2D human subjects, as well as in obese mice. Hence, the various VGF peptide immunoreactivities we studied appeared to be downregulated with obesity, as well as in T2D human subjects with or without obesity. Our results strongly argue for a key role of VGF peptides in mechanisms related to energy balance and glucose metabolism, as recent studies indicate. Chronic injection of TLQP-21 in pre-diabetic ZDF rats preserved islet cell mass and slowed the onset of diabetes [17]. More recently, NERP-2 stimulated food intake when injected *i*.*c*.*v*. [36], while increasing glucose induced insulin secretion upon i.v. administration [19]. As to the source of circulating VGF peptides, the endocrine pancreas appears to be a major site of expression of VGF peptides [28, 31]. Other endocrine and/or neuroendocrine sources are likely to contribute plasma VGF peptides, including at least adrenal gland [25], pituitary [26] and ovary [33], though possible changes in VGF peptide profiles upon obesity and/or diabetes in such locations are as yet undetermined.

### VGF peptides in mouse tissues

As for plasma, tissue VGF immunoreactive peptide levels in BAT and WAT were higher in slim *vs*. obese mice, and/or showed a distinct response to glucose in the former, but not in the latter. Some differences were revealed, the glucose load leading to a striking increase in tissue concentrations of VGF C-terminus, TLQP and NAPPE peptides in WAT, while the response was lower and confined to VGF C-terminus peptide/s in BAT. On such basis, one could speculate that VGF peptides may be involved in different mechanisms in such two locations.

VGF peptides are widely present in sympathetic pathways, encompassing the hypothalamic nuclei [35, 37], as well as the celiac ganglion, WAT, BAT and in sympathetic axons and terminals innervating pancreatic islets, as shown here. The prompt response of both TH and VGF peptides upon the glucose load in sympathetic axons supplying pancreatic islets, together with their increase in the peripheral circulation, suggests a distinct increase of VGF peptide biosynthesis and transport to terminals, together with stimulation of their release, arguing for a likely dynamic regulatory role in response to metabolic stimuli.

In our small series of patients, VGF peptides and/or their response to glucose were down regulated in both T2D patients and euglycemic obese subjects. Since obesity often precedes, and increases the risk of T2D, it will be of interest to investigate the development of changes in VGF peptide profiles in human obesity, as a potential clue to the their role/s in the development of T2D, and/or as a lead to the development of novel monitoring or therapeutic strategies aimed at controlling obesity and/or the evolution to overt T2D.

At the same time, it ought to be noticed that the expression of VGF mRNA and/or peptides in adipocytes, and/or in some of migratory and inflammatory cells localized within the adipose tissue awaits full clarification. While local release of VGF peptides from certain tissues components (e.g. sympathetic terminals) may be reflected in plasma changes due to a wash-out effect, possible release from other sources to act *via* a hormonal pathway ought to be considered.

### Molecular heterogeneity, actions and role/s of VGF peptides

As mentioned, the immunoreactivities we measured are expected to include different molecular forms of the VGF peptides studied. Differential activities of differing molecular species must be considered, as well as differences in VGF and its derived peptides across species, such as shown for the C-terminal domain of VGF. TLQP-21 increased resting energy expenditure upon *i*.*c*.*v*. injection, *via* up-regulation of β2 adrenergic receptors in BAT and UCP1 in WAT [14]. It also showed stimulatory action on lipolysis in WAT [15], as well as enhancing insulin secretion and glucose tolerance [17]. The C-terminally extended peptide TLQP-62, which would be detected by both the TLQP and the VGF C-terminus assay and antibodies in the present study, proved more powerful in stimulating insulin secretion from insulinoma cell lines, compared to TLQP-21 and several other fragments from the C-terminal domain of VGF, and improved glucose tolerance when injected peripherally in mice [18]. Conversely, a study on the isolated perfused rat pancreas failed to confirm the stimulatory action of TLQP-21 on insulin secretion [38]. Depending on the actual balance of TLQP-21 and TLQP-62 peptides, one could hypothesize a decreased availability TLQP-21 releasable from sympathetic terminals in WAT. Together with the increased binding capacity for TLQP-21 found in WAT of diet induced obese mice [15], such a decreased availability would point to a pathophysiological mechanism of altered WAT regulation in obesity, further broadening the multifaceted impact of diabetic neuropathy [39]. Conversely, a reduction of both TLQP-62 and TLQP-21 could result in defective stimulation on insulin secretion from pancreatic islets. Recently, at least one receptor for TLQP-21 has been identified [40] and characterized in its precise reactivity depending on the precise C-terminal sequence of the TLQP-21 peptide [16]. In such region, the human sequence shows some difference from the rat/mouse sequence, resulting in an approximately 20% potency on the rat receptor [16]. A further sequence difference in a near by region appears to result in a C-terminally amidated TLQP-24 form, while the rat/mouse VGF does not show the corresponding cleavage and C-terminal amidation site. Human TLQP-24 showed anti-bacterial activity, but its role *in vivo* is so far unknown [41]. As to The QQET-30 (NERP-3) and NAPP-19 (NERP-4) peptides, little is so far known as to their action and possible roles in the present context. Recently, the expression in mouse of human VGF, or a C-terminally deleted form (93 amino acids) resulted in increased, or decreased body weight, respectively [42]. While the differing human *vs*. mouse VGF sequences may well result in functional differences, one or more peptides deriving from the C-terminal domain of VGF are likely to be endowed with critical role/s in adiposity and energy balance regulation.

## Supporting Information

S1 Fig NAPP-19 HPLC-ESI-MS/MS spectrum. (DOC)

S2 Fig Human-VGF (487–503) HPLC-ESI-MS/MS spectrum. (DOC)

S1 Dataset Human plasma dataset. (JPG)

S2 Dataset Mouse tissue dataset. (JPG)
