# Supplementary figures and images for: VGF Peptide Profiles in Type 2 Diabetic Patients’ Plasma and in Obese Mice
Source: PLoS One. 2015 Nov 12;10(11):e0142333. doi: 10.1371/journal.pone.0142333 (PMC4643017; doi:10.1371/journal.pone.0142333)

(NAPP-19) NAPPEPVPPPRAAPAPTHV

| **MH+1(av)** | **MH+1(mono)** |
| --- | --- |
| 1916.2036 | 1915.0185 |

**Theoretical Peak Table**

| **b** |  |  |  | **y** |
| --- | --- | --- | --- | --- |
|  |  |  |  |  |
| --- | 1 | **N** | 19 | --- |
| 186.0873 | 2 | **A** | 18 | 1800.9755 |
| 283.1401 | 3 | **P** | 17 | 1729.9384 |
| 380.1928 | 4 | **P** | 16 | 1632.8857 |
| 509.2354 | 5 | **E** | 15 | 1535.8329 |
| 606.2882 | 6 | **P** | 14 | 1406.7903 |
| 705.3566 | 7 | **V** | 13 | 1309.7375 |
| 802.4094 | 8 | **P** | 12 | 1210.6691 |
| 899.4621 | 9 | **P** | 11 | 1113.6164 |
| 996.5149 | 10 | **P** | 10 | 1016.5636 |
| 1152.6160 | 11 | **R** | 9 | 919.5108 |
| 1223.6531 | 12 | **A** | 8 | 763.4097 |
| 1294.6902 | 13 | **A** | 7 | 692.3726 |
| 1391.7430 | 14 | **P** | 6 | 621.3355 |
| 1462.7801 | 15 | **A** | 5 | 524.2827 |
| 1559.8329 | 16 | **P** | 4 | 453.2456 |
| 1660.8806 | 17 | **T** | 3 | 356.1928 |
| 1797.9395 | 18 | **H** | 2 | 255.1452 |
| --- | 19 | **V** | 1 | 118.0863 |


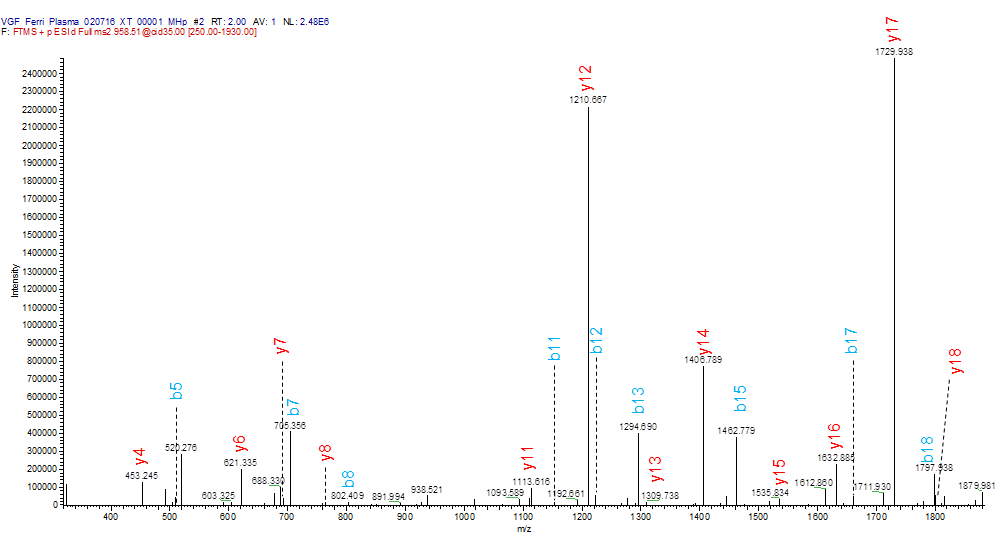

Supplement: S1 Fig — (DOC) [file pone.0142333.s001.doc]

**Human-VGF (487-503) PPEPVPPPRAAPAPTHV**

| **MH+1(av)** | **MH+1(mono)** |
| --- | --- |
| 1731.0198 | 1729.9384 |

**Theoretical Peak Table**

| **b** |  |  |  | **y** |
| --- | --- | --- | --- | --- |
|  |  |  |  |  |
| --- | 1 | **P** | 17 | --- |
| 195.1128 | 2 | **P** | 16 | 1632.8857 |
| 324.1554 | 3 | **E** | 15 | 1535.8329 |
| 421.2082 | 4 | **P** | 14 | 1406.7903 |
| 520.2766 | 5 | **V** | 13 | 1309.7375 |
| 617.3293 | 6 | **P** | 12 | 1210.6691 |
| 714.3821 | 7 | **P** | 11 | 1113.6164 |
| 811.4349 | 8 | **P** | 10 | 1016.5636 |
| 967.5360 | 9 | **R** | 9 | 919.5108 |
| 1038.5731 | 10 | **A** | 8 | 763.4097 |
| 1109.6102 | 11 | **A** | 7 | 692.3726 |
| 1206.6630 | 12 | **P** | 6 | 621.3355 |
| 1277.7001 | 13 | **A** | 5 | 524.2827 |
| 1374.7528 | 14 | **P** | 4 | 453.2456 |
| 1475.8005 | 15 | **T** | 3 | 356.1928 |
| 1612.8594 | 16 | **H** | 2 | 255.1452 |
| --- | 17 | **V** | 1 | 118.0863 |
|  |  |  |  |  |


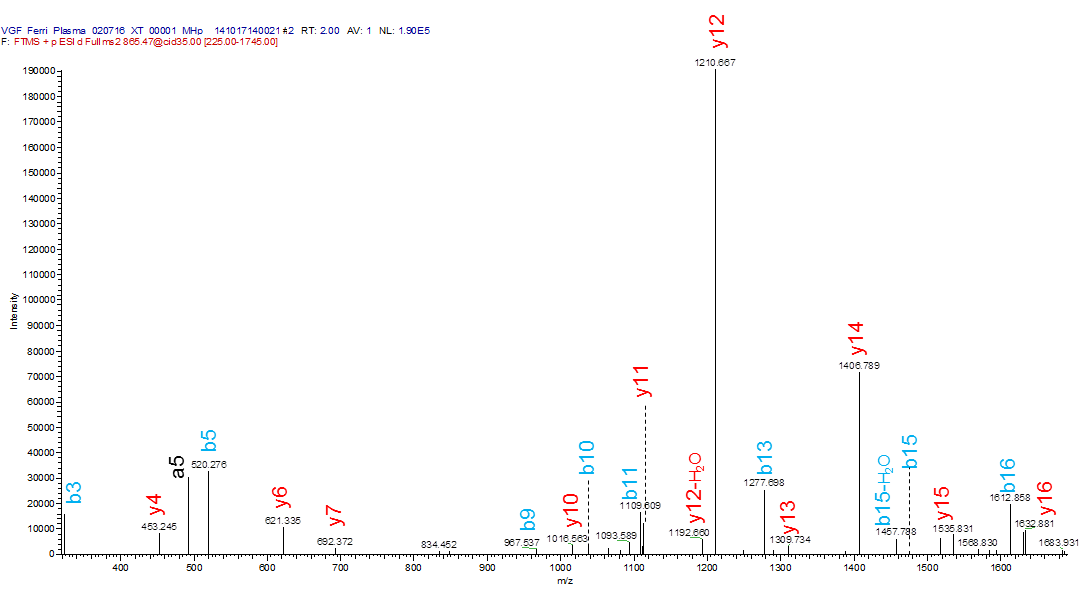

Supplement: S2 Fig — (DOC) [file pone.0142333.s002.doc]

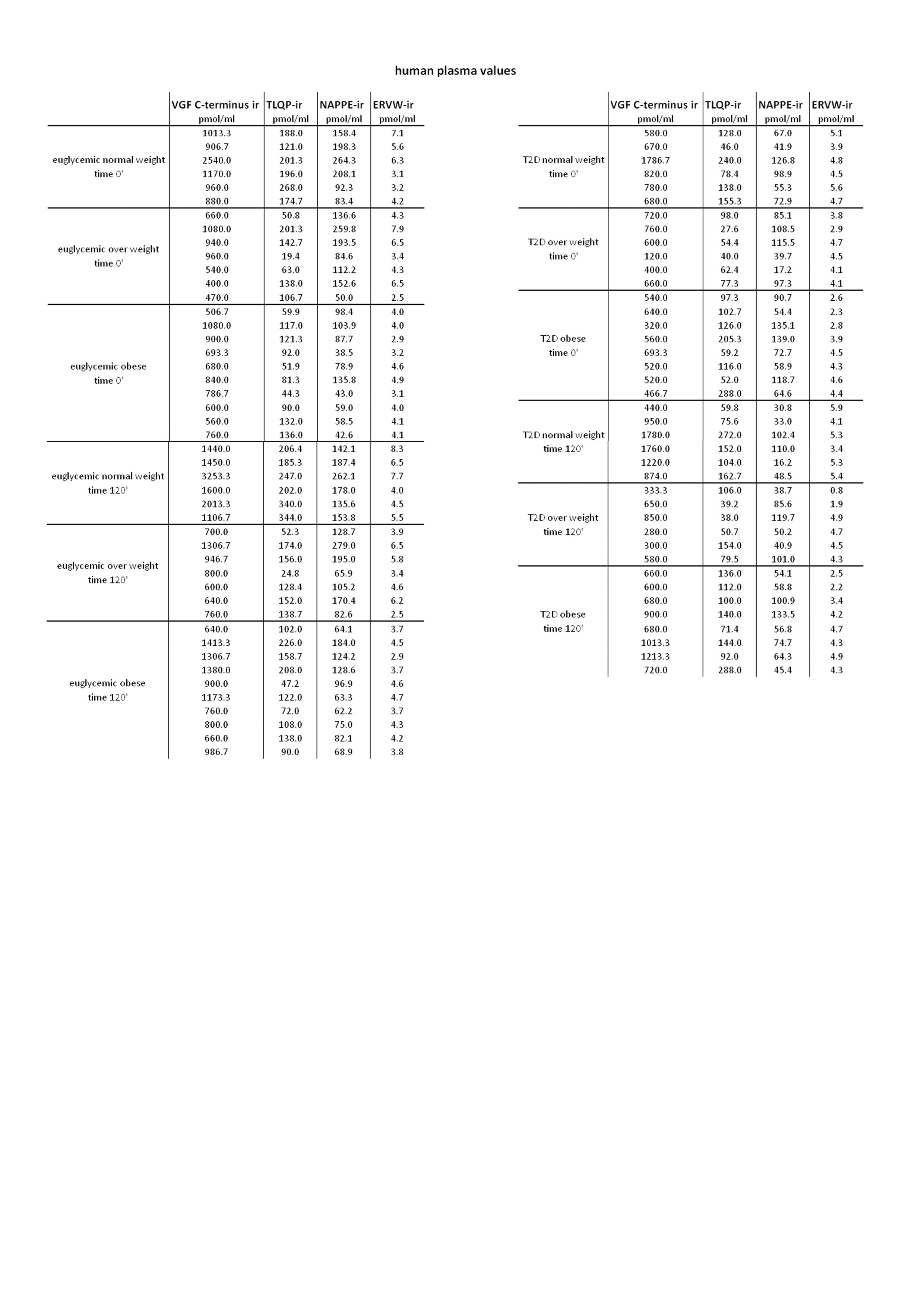

Supplement: S1 Dataset — (JPG) [file pone.0142333.s003.jpg]

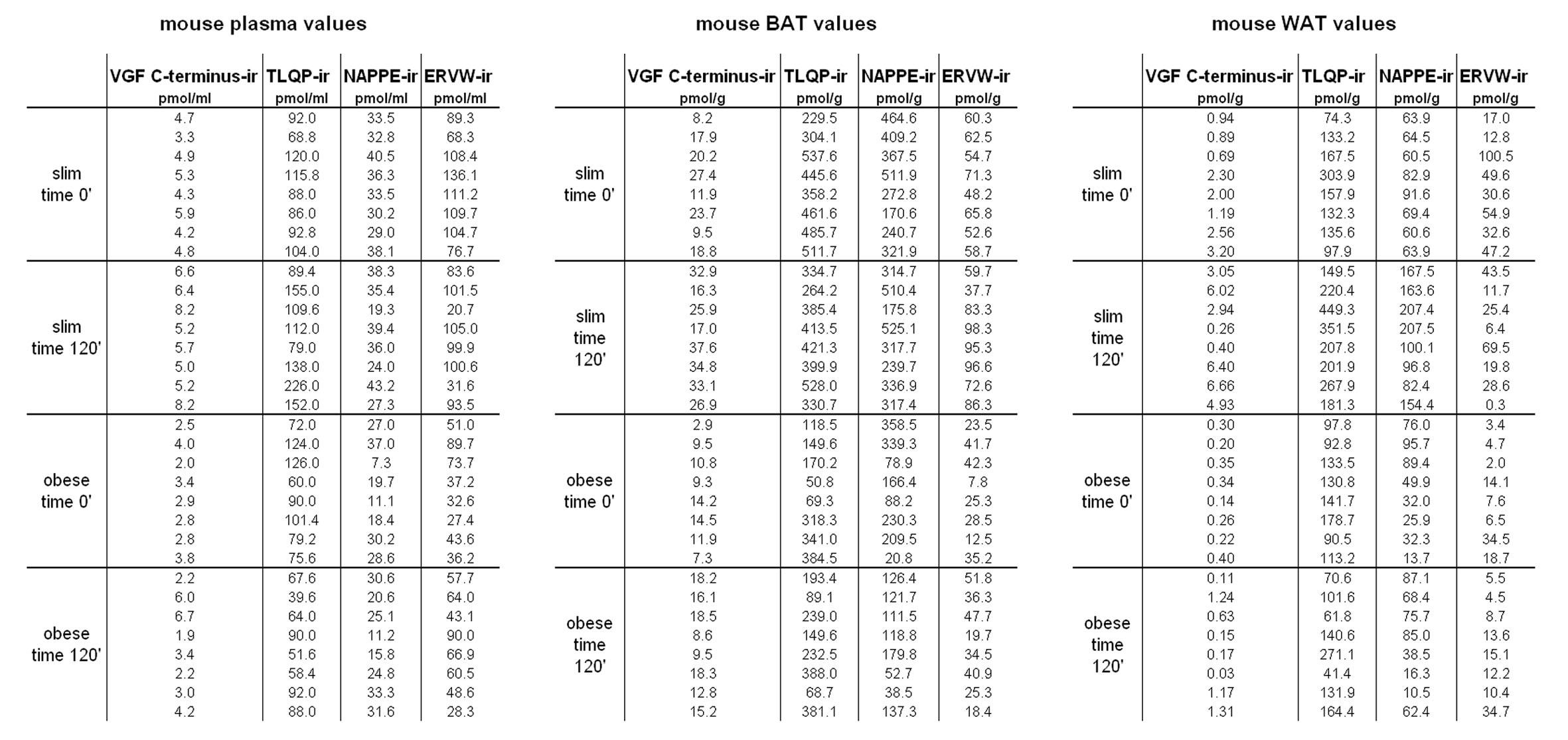

Supplement: S2 Dataset — (JPG) [file pone.0142333.s004.jpg]
